# Phylogeography of the Crown-of-Thorns Starfish in the Indian Ocean

**DOI:** 10.1371/journal.pone.0043499

**Published:** 2012-08-21

**Authors:** Catherine Vogler, John Benzie, Paul H. Barber, Mark V. Erdmann, Ambariyanto, Charles Sheppard, Kimberly Tenggardjaja, Karin Gérard, Gert Wörheide

**Affiliations:** 1 Department für Geo- und Umweltwissenschaften and GeoBio-CenterLMU, Ludwig-Maximilians-Universität München, München, Germany; 2 Environmental Research Institute, University College Cork, Cork, Ireland; 3 Department of Ecology and Evolutionary Biology, University of California Los Angeles, Los Angeles, California, United States of America; 4 Conservation International, Indonesia Marine Program, Bali, Indonesia; 5 Faculty of Fisheries and Marine Science, Diponegoro University, Semarang, Indonesia; 6 Department of Biological Sciences, University of Warwick, Coventry, United Kingdom; 7 Ecology and Evolutionary Biology Department, University of California Santa Cruz, Santa Cruz, California, United States of America; 8 Université de la Méditerranée, Station Marine d’Endoume, Marseille, France; 9 Bavarian State Collections of Palaeontology and Geology, München, Germany; Biodiversity Insitute of Ontario - University of Guelph, Canada

## Abstract

**Background:**

Understanding the limits and population dynamics of closely related sibling species in the marine realm is particularly relevant in organisms that require management. The crown-of-thorns starfish *Acanthaster planci,* recently shown to be a species complex of at least four closely related species, is a coral predator infamous for its outbreaks that have devastated reefs throughout much of its Indo-Pacific distribution.

**Methodology/Principal Findings:**

In this first Indian Ocean-wide genetic study of a marine organism we investigated the genetic structure and inferred the paleohistory of the two Indian Ocean sister-species of *Acanthaster planci* using mitochondrial DNA sequence analyses. We suggest that the first of two main diversification events led to the formation of a Southern and Northern Indian Ocean sister-species in the late Pliocene-early Pleistocene. The second led to the formation of two internal clades within each species around the onset of the last interglacial. The subsequent demographic history of the two lineages strongly differed, the Southern Indian Ocean sister-species showing a signature of recent population expansion and hardly any regional structure, whereas the Northern Indian Ocean sister-species apparently maintained a constant size with highly differentiated regional groupings that were asymmetrically connected by gene flow.

**Conclusions/Significance:**

Past and present surface circulation patterns in conjunction with ocean primary productivity were identified as the processes most likely to have shaped the genetic structure between and within the two Indian Ocean lineages. This knowledge will help to understand the biological or ecological differences of the two sibling species and therefore aid in developing strategies to manage population outbreaks of this coral predator in the Indian Ocean.

## Introduction

A growing body of research shows that cryptic speciation is common in the marine realm (reviewed in [1], [2]). Indeed, molecular genetic surveys of natural populations are increasingly identifying sibling species, closely related sister-species which are often *a priori* morphologically indistinguishable and are thus classified as a single nominal species [3]. This is even the case in widespread marine organisms with long-lived pelagic larvae that could be expected to display little genetic structure [1]. Identifying closely related sibling species and the processes that drive their speciation is essential to understanding evolutionary processes in the marine environment and can shed light on the importance of past and present barriers to gene flow in marine systems [4].

Understanding the extent of the genetic differences between sister-species is especially important in organisms where the presence of sibling species could have far-reaching impacts, such as biological model organisms, commercially valuable species, biological indicator species or organisms that require management, such as threatened species and pests [3], [5]. The corallivorous crown-of-thorns starfish (COTS) *Acanthaster planci* is of particular interest in this regard as it undergoes population outbreaks that have devastated coral reefs throughout much of its distribution range since the 1960s [6]. Although outbreaks still account for a large proportion of the disturbance to Indo-Pacific reefs today [7], the causes of these outbreaks and appropriate monitoring strategies to predict their occurrence and management plans to reduce their impact are still debated [6]–[9].

Once thought to be a single species, research by Vogler et al. [10] showed that the crown-of-thorns starfish is a species complex comprised of four highly differentiated evolutionary lineages with restricted ranges located in (i) the Pacific, (ii) the Red Sea, (iii) the northern and (iv) the southern Indian Ocean. Phylogenetic analysis indicates that the northern and southern Indian Ocean clades are closely related sister groups, to the exclusion of the Red Sea and Pacific clades, which also formed a clade, albeit with low statistical support [10].

As an important and destructive predator on coral reefs, many studies have examined the ecology and population dynamics in *A. plancii* (e.g., [11], [12],[8],[13],[14]). However, the overwhelming majority of COTS research has been performed on the Pacific species under the assumption that these populations were representative of the entire range. The failure to recognise the existence of a species complex and extrapolation of Pacific COTS studies to the entire distribution of COTS for both research and management purposes may thus mask potentially important ecological differences among geographically unique lineages, contributing to a lack of understanding of the processes that lead to regional outbreaks in the different COTS lineages [10]. Indeed, although outbreaks are also a reason for concern in the Indian Ocean [12], [13] and the Red Sea [15], they do not appear to be as massive or widespread as in the Pacific [16], a pattern that might be indicative of key biological or ecological differences between the sister-species.

Previous genetic studies on COTS populations have largely focused on the genetic differences among Pacific and Indian Ocean lineages, and have included limited geographic sampling from the Indian Ocean [11], [14], [17]. In this study, we conduct a basin-wide examination of COTS’ population genetic structure within the Indian Ocean to 1.) identify the geographic distributions of the Northern and Southern Indian Ocean COTS lineages and gain a better understanding of the processes that led to the diversification of these two sister-species in the Indian Ocean, and 2.) explore differences in long-term population dynamics that may have resulted from biological or ecological differences among the two sibling species.

## Results

### Sampling and Sequencing

Of the 190 samples for which we obtained mitochondrial putative control region (CR) sequences, 95 belonged to the Northern Indian Ocean (NIO) sister-species (522 bp) and 95 to the Southern Indian Ocean (SIO) sister-species (546 bp; Table 1, Table S1). The corresponding mitochondrial partial cytochrome oxidase subunit I gene (COI) dataset (632 bp) included 48 individuals of the NIO sister-species, and 57 of the SIO sister-species (Table 1, Table S1). Haplotype and nucleotide diversities were high for the CR datasets, and lower for the COI dataset (Table 1; see Table 2 and 3 for population level statistics). The COI dataset was thus more appropriate for interspecific analyses, and the CR datasets for intraspecific analyses.

**Table 1:** Summary statistics per sister-species and dataset.

Dataset	Sequence length (bp)	*n*	*h*_D_	*Π*	*F*_S_	*D*	*R*_2_
**Northern Indian Ocean sister-species**							
COI	632	48	0.68 (±0.045)	0.004 (±0.0059)	−1.32	−0.13	0.101
CR	522	95	0.98 (±0.006)	0.020 (±0.0102)	**−24.44**	−0.28	0.079
**Southern Indian Ocean sister-species**							
COI	632	57	0.59 (±0.074)	0.002 (±0.0013)	**−12.67**	**−2.08**	**0.036**
CR	546	95	0.99 (±0.003)	0.016 (±0.0082)	**−24.65**	**−1.57**	**0.050**

COI: Cytochrome Oxidase I and CR: Control Region; bp, aligned sequence length; *n,* number of individuals; *h*_D_, haplotype diversity; *π*, nucleotide diversity; Fu’s *F*_S_; Tajima’s *D*; Ramos-Onsins *R*_2_; significant values are bold.

**Table 2:** AMOVA results for the Southern and Northern Indian Ocean sister-species.

	Northern Indian Ocean sister-species	Southern Indian Ocean sister-species
	*west* vs. *central* vs. *east* 1	*prov19* vs. *prov20* vs. *prov22* vs. *prov27* 2
Overall Φ_CT_ (between groups)	0.574***	0.056*
Overall Φ_SC_ (within groups)	0.066***	0.025**
Percent variation:		
Among groups	57.37%	5.64%
Among populations within groups	2.82%	2.36%
Within populations	39.81%	92.00%

1 *west*: UAE, Oman; *central*: Maldives; *east*: Thailand, Aceh, Christmas Island, Pulau Seribu, Krakatau, Karimunjawa.

2 *prov19*: UAE, Oman; *prov20*: Kenya, South Africa, Mayotte, South Madagascar, North Madagascar, Réunion, Mauritius; *prov22*: Chagos; *prov27*: Cocos Keeling Islands.

Significance tested with 50,000 permutations; *p<0.05, **p<0.01 and ***p<0.001.

**Table 3:** Summary statistics per location based on the Control Region dataset.

Location	*n*	*h*_T_	*h*_P_	*h*_F_	*h*	*π*	*F*_S_	*D*	*R*_2_
**Southern Indian Ocean** **sister-species**									
UAE	2	2	1	0.50	1.00 (±0.500)	0.004 (±0.0045)	0.69	–	0.500
Oman	2	2	1	0.50	1.00 (±0.500)	0.032 (±0.0325)	2.83	–	0.500
Reunion	5	5	2	0.40	1.00 (±0.127)	0.011 (±0.0075)	−1.06	−0.28	0.138
Mauritius	4	4	4	1.00	1.00 (±0.177)	0.010 (±0.0074)	−0.40	−0.07	0.137
Kenya	24	22	17	0.77	0.99 (±0.014)	0.017 (±0.0090)	**−11.82**	−1.29	0.077
South Africa	12	12	11	0.92	1.00 (±0.034)	0.017 (±0.0094)	**−5.33**	**−1.58**	0.099
Mayotte	21	19	15	0.79	0.99 (±0.018)	0.015 (±0.0082)	**−9.55**	−1.17	0.081
Nth Madagascar	11	11	9	0.82	1.00 (±0.039)	0.017 (±0.0098)	**−4.40**	−1.38	**0.091**
Sth Madagascar	2	2	2	1.00	1.00 (±0.500)	0.043 (±0.0436)	3.14	–	0.500
Chagos	6	6	5	0.83	1.00 (±0.096)	0.016 (±0.0091)	−1.23	−1.14	**0.055**
Cocos Keeling Islands	6	3	2	0.67	0.73 (±0.155)	0.003 (±0.0024)	0.54	−0.93	0.373
**Northern Indian Ocean** **sister-species**									
UAE	15	11	7	0.64	0.95 (±0.040)	0.004 (±0.0029)	**−5.67**	−0.60	**0.084**
Oman	9	6	2	0.33	0.89 (±0.091)	0.005 (±0.0032)	−1.66	−0.77	0.123
Maldives	17	15	12	0.80	0.99 (±0.025)	0.018 (±0.0097)	**−5.51**	0.07	0.119
Christmas Island	3	1	0	0.00	0.00 (±0.000)	0.000 (±0.0000)	–	–	–
Aceh	15	11	8	0.73	0.93 (±0.054)	0.012 (±0.0068)	−2.48	−1.13	0.093
Thailand	16	14	8	0.57	0.98 (±0.028)	0.010 (±0.0057)	**−7.55**	−0.45	0.116
Pulau Seribu	12	10	6	0.60	0.97 (±0.044)	0.011 (±0.0063)	−3.21	−0.49	0.122
Karimunjawa	5	4	3	0.75	0.90 (±0.161)	0.012 (±0.0083)	0.88	−0.35	0.180
Krakatau	3	3	2	0.67	1.00 (±0.272)	0.018 (±0.0144)	1.07	–	0.205
**Location**	***n***	***h*** **_T_**	***h*** **_P_**	***h*** **_F_**	***h*** **_D_**	***π***	***F*** **_S_**	***D***	***R*** **_2_**
**Southern Indian Ocean** **sister-species**									
UAE	2	2	1	0.50	1.00 (±0.500)	0.004 (±0.0045)	0.69	–	0.500
Oman	2	2	1	0.50	1.00 (±0.500)	0.032 (±0.0325)	2.83	–	0.500
Reunion	5	5	2	0.40	1.00 (±0.127)	0.011 (±0.0075)	−1.06	−0.28	0.138
Mauritius	4	4	4	1.00	1.00 (±0.177)	0.010 (±0.0074)	−0.40	−0.07	0.137
Kenya	24	22	17	0.77	0.99 (±0.014)	0.017 (±0.0090)	**−11.82**	−1.29	0.077
South Africa	12	12	11	0.92	1.00 (±0.034)	0.017 (±0.0094)	**−5.33**	−**1.58**	0.099
Mayotte	21	19	15	0.79	0.99 (±0.018)	0.015 (±0.0082)	**−9.55**	−1.17	0.081
Nth Madagascar	11	11	9	0.82	1.00 (±0.039)	0.017 (±0.0098)	**−4.40**	−1.38	**0.091**
Sth Madagascar	2	2	2	1.00	1.00 (±0.500)	0.043 (±0.0436)	3.14	–	0.500
Chagos	6	6	5	0.83	1.00 (±0.096)	0.016 (±0.0091)	−1.23	−1.14	**0.055**
Cocos Keeling Islands	6	3	2	0.67	0.73 (±0.155)	0.003 (±0.0024)	0.54	−0.93	0.373
**Northern Indian Ocean** **sister-species**									
UAE	15	11	7	0.64	0.95 (±0.040)	0.004 (±0.0029)	**−5.67**	−0.60	**0.084**
Oman	9	6	2	0.33	0.89 (±0.091)	0.005 (±0.0032)	−1.66	−0.77	0.123
Maldives	17	15	12	0.80	0.99 (±0.025)	0.018 (±0.0097)	**−5.51**	0.07	0.119
Christmas Island	3	1	0	0.00	0.00 (±0.000)	0.000 (±0.0000)	–	–	–
Aceh	15	11	8	0.73	0.93 (±0.054)	0.012 (±0.0068)	−2.48	−1.13	0.093
Thailand	16	14	8	0.57	0.98 (±0.028)	0.010 (±0.0057)	**−7.55**	−0.45	0.116
Pulau Seribu	12	10	6	0.60	0.97 (±0.044)	0.011 (±0.0063)	−3.21	−0.49	0.122
Karimunjawa	5	4	3	0.75	0.90 (±0.161)	0.012 (±0.0083)	0.88	−0.35	0.180
Krakatau	3	3	2	0.67	1.00 (±0.272)	0.018 (±0.0144)	1.07	−	0.205

*n,* number of individuals; *h*_T_, total number of haplotypes; *h*_P_, number of private haplotypes; *h*_F_, private haplotype frequency; *h*_D_, haplotype diversity; *π*, nucleotide diversity; Fu’s *F*_S_; Tajima’s *D*; Ramos-Onsins *R*_2_; significant values are bold.

### Divergence Times and Demographic Patterns

The time of divergence between the two Indian Ocean sister-species was estimated to be 1.86–2.89 Mya, in the late Pliocene-early Pleistocene based on the net divergence *d*_A_ of the K2P distances from the COI dataset (divergence rate: 3.7±0.8%.Myr-1; [18]).

**Figure 1:**
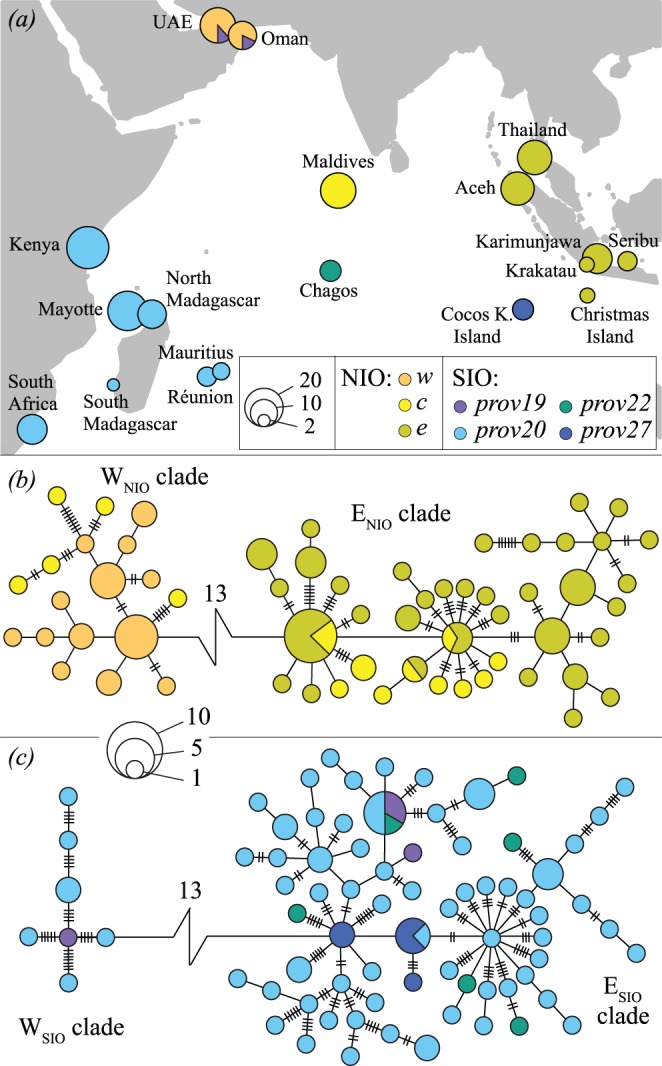
Phylogeography of the crown-of-thorns starfish in the Indian Ocean. *(a)* sampling locations from the Northern and Southern Indian Ocean sister-species (here denoted as NIO and SIO respectively), circles are proportional to sample size, colours indicate the regional grouping of populations that explained most of the variance amongst groups (NIO: *w*: *west, c: central, e: east*; SIO: *prov19*, *20*, *22*, *27* =  Marine ecoregions regional provinces (Marine Ecoregions of the World: http://www.worldwildlife.org/science/ecoregions/marine/provinces.htm; [19])). *(b)* and *(c)* Minimum spanning trees (CR) of NIO and SIO respectively, all haplotypes are separated by one mutational step unless denoted by a higher number of hatch marks, except the clades W_NIO_ and E_NIO_ as well a W_SIO_ and E_SIO_ which are separated by 13 mutational steps. Colours are the same as in *(a)* and circle size is proportional to frequency of occurrence.

Minimum spanning trees for each sister-species showed two clades separated by a large internal split of 13 mutation steps. In the NIO sister-species, one clade consisted of CR haplotypes found only in the west and central northern Indian Ocean sites (here called W_NIO_), and the other consisted of CR haplotypes found only in the eastern and central northern Indian Ocean (E_NIO_; Fig. 1). In the SIO sister-species, one clade consisted of CR haplotypes found only in western Indian Ocean sites (W_SIO_), the second consisted of CR haplotypes spread throughout the southern Indian Ocean but apparently derived from ancestors found in Cocos Keeling Islands, thus of eastern origin (E_SIO_; Fig. 1). These clades and the central position of the Cocos Keeling CR haplotypes were also recovered in the NeighborNets (Fig. S1), supporting the robustness of this signal. The net divergence *d*_A_ between these clades was similar: 3.98% for W_NIO_ vs. E_NIO_, and 3.50% for W_SIO_ vs. E_SIO_, as were the *T*_MRCA_s for each lineage: 139’600 years ago for the NIO sister-species, 113’700 for the SIO sister-species.

The Bayesian skyline plots showed some signs of recent expansions in some populations of both sister-species, potentially indicating an expansion after the last glacial maximum (18,000–24,000 years ago), but in both cases a very large variance around the parameter estimates limited the interpretability of the data (Fig. S2). However, all other demographic statistics showed no signs of a recent population expansion for the NIO sister-species (*F*_s_, *D*, and *R*_2_ not significant except *F*_s_ estimated with the CR dataset; Table 1) whereas the SIO sister-species clearly did (*F*_s_, *D*, and *R*_2_ significant for both COI and CR; Table 1, see also Table 2 and 3).

### Spatial Genetic Structure and Migration Patterns

The overall Φ_ST_ of the NIO sister-species without *a priori* structure was strong (Φ_ST_ = 0.51, p<0.001), whereas structure in the SIO sister-species was weak (Φ_ST_ = 0.07, p<0.001). Indeed, 14 of the 36 pairwise Φ_ST_ comparisons in the NIO sister-species were significant after Bonferroni correction, whereas none of the 55 SIO sister-species comparisons were (Table S4). There was significant isolation by distance in the NIO sister-species as revealed by the positive regression between Φ_ST_/(1− Φ_ST_) and the logarithm of geographic distances (*b* = 1.28, *R* ^2^ = 0.35, *p*<0.001; Fig. S3a), and no relationship in the SIO sister-species (*b* = 0.11, *R* ^2^ = 0.06, *p*>0.05; Fig. S3b).

According to the AMOVA analyses, the regional groupings explaining most of the genetic variation in the NIO sister-species were composed of a western group (*west*: Oman and UAE), a central group (*central*: Maldives) and an eastern group (*east*: Thailand, Aceh, Christmas Island, Pulau Seribu, Krakatau and Karimunjawa; Fig. 1). In the SIO sister-species, they followed the Marine Ecoregions of the World provinces [19]: province 19 (*prov19*: Oman and UAE), province 20 (*prov20*: Kenya, Mayotte, North Madagascar, South Madagascar, South Africa, Réunion and Mauritius), province 22 (*prov22*: Chagos) and province 27 (*prov27*: Cocos Keeling Islands; Fig. 1). In the NIO sister-species, most of the genetic variation was explained among regional groups (57.37%, Φ_CT_ = 0.574, p<0.001) within which variation was low (2.82%, Φ_SC_ = 0.066, p<0.001; Table 2). In the SIO sister-species, although we present the regional combination that maximised genetic variation among groups, this explained little of the total variation (5.64%, Φ_CT_ = 0.056, p<0.05), with most of the variation occurring between individuals within populations (92%; Table 2).

**Figure 2:**
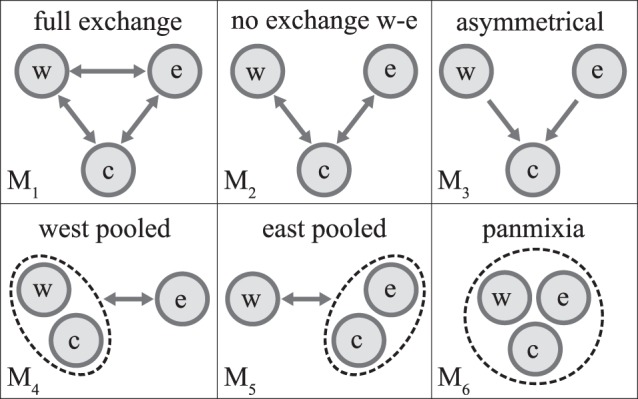
Migration models compared in the Migrate analysis of the Northern Indian Ocean sister-species. Migration models range from M_1_: full exchange to M_6_: panmixia. *west* (w), *east* (e) and *central* (c) represent the regional groupings displayed in Fig. 1; arrows indicate direction of migration.

Migrate analyses based on the regional groupings identified by the AMOVA were initially run with a full exchange matrix (i.e., bidirectional exchange of migrants possible between all regional groups) to determine appropriate priors (Table S3). The process was straightforward for the NIO sister-species, but the chains did not converge for the SIO sister-species, even in very long runs. Since the groupings in the SIO sister-species were of unequal sizes, we restricted the analysis to the larger populations, i.e. within *prov20* only (excl. South Madagascar). However, the only model that converged was the panmixia model, suggesting gene flow was too high within this province to determine individual migration rates between populations and allow a proper comparison of migration models, a result consistent with the minimal CR genetic structure in this species. Therefore, we only present the Migrate results for the NIO sister-species where a series of different migration models could be tested (Fig. 2).

Log Bayes factors indicate strong support [20] in favour of the asymmetrical migration model M_3_, allowing migration from the regional groups *west* and *east* towards *central* but not back to these groups or between them (Table 4). For this model, the effective number of migrants per generation (Ne_i_m_j→i_ = Θ_i_*M_j→i_) from *west* to *central* was 218, and from *east* to *central* 254 (Table 5). The second best ranking model, the full exchange model M_1_, essentially revealed the same migration patterns as M_3_ but with a stronger contribution to *central*’s gene pool from *east* than from *west* (Table 5).

**Table 4:** Performance of different gene flow models between regional groupings in the Northern Indian Ocean sister-species (Fig. 2), ranked against M_3_, the best-performing model.

Model	*l*_M_	LBF	Rank
M_1_	−1954.69	−13.4	2
M_2_	−1975.56	−34.3	3
M_3_	−1941.29	0.0	1
M_4_	−1995.19	−53.9	5
M_5_	−1991.02	−49.7	4
M_6_	−2031.98	−90.7	6

*l*_M_: Log marginal likelihood, LBF: Log Bayes factors.

**Table 5:** Migration matrix of the two most supported gene flow models in the Northern Indian Ocean sister-species (M_3_ and M_1_; Fig. 2).

from/to	*west*	*central*	*east*
***west***	0.015	654 (218)	0 (0)
	0.010	178 (68)	23 (0.9)
***central***	0 (0)	0.333	0 (0)
	60 (0.5)	0.379	77 (3)
***east***	0 (0)	762 (254)	0.015
	56 (0.5)	693 (262)	0.038

Θ_i_ (diagonal) and the number of migrants from regional grouping i to j per generation, followed by the migration rates in brackets. Top numbers are the results for the asymmetrical model M_3_, bottom numbers for the full exchange model M_1_.

## Discussion

Previous genetic studies of *Acanthaster planci* have focused on highlighting differences among Indian and Pacific Ocean populations using a limited number of Indian Ocean samples [11], [14], [17]. However, increased sampling of the Indian Ocean basin revealed the presence of sibling species within the Indian Ocean [10], and rapidly evolving mtDNA control region sequence data also indicates significant genetic structure within these sibling species. The Northern Indian Ocean sister-species, ranging from the shores of Indonesia to the Gulf of Oman, showed strong genetic structure (Φ_ST_ = 0.51, p<0.001) between western (Oman and UAE), central (Maldives) and eastern populations (Thailand, Aceh, Christmas Island, Pulau Seribu, Krakatau and Karimunjawa) (Fig. 1). In contrast, in the Southern Indian Ocean sibling species (Oman, UAE, Kenya, Mayotte, Madagascar, South Africa, Réunion, Mauritius, Chagos, Cocos Keeling; Fig. 1), structure was much weaker (Φ_ST_ = 0.07, p<0.001).

The recovery of distinct Indian Ocean lineages highlighted the presence of barriers to genetic exchange within this ocean basin, even though there are no obvious barriers to dispsersal and COTS have relatively long pelagic larval durations of 3–4 weeks, based on research from Pacific COTS [21]. That these two distinct evolutionary lineages have radically different levels of genetic structure across areas of the Indian Ocean without obvious barriers to dispersal, despite having very similar geographic ranges, strongly suggests that they are either impacted by different environmental processes that shape connectivity and dispersal across their range, or have unique ecological or biological characters that influence their dispersal and connectivity. These abiotic and biotic variables, either singly or in concert, then drive differential evolutionary processes in the two species.

### Diversification Processes

The application of a molecular clock suggests that the diversification of the Northern (NIO) and the Southern Indian Ocean (SIO) sister-species of the crown-of-thorns starfish occurred during the late Pliocene-early Pleistocene (1.86–2.89 Mya). Although the exact timing of this event should be interpreted with caution, as no external calibration points were available and the mutation rate we used was inferred from other echinoderms (Echinoidea; [18]), this general period coincides with periods of strong climatically-induced sea-level fluctuations. Indeed, global sea levels repeatedly dropped 120 m below their present level during glaciations in the early Pleistocene (2.5, 2.2, 2.1 and 1.9 Mya; [22]).

Sea-level changes have frequently been invoked as a driver of speciation on coral reefs [23], [24], particularly among Pacific and Indian Ocean populations (for reviews, see [25], [26]), because the dominant mode of speciation is allopatric [27], [28] and there are few obvious allopatric barriers in the sea [23], [29]. Low sea-level stands during glacial periods are thought to have restricted dispersal pathways and/or altered the distribution of reef-dwelling organisms [30], promoting evolutionary diversification. As NIO and SIO COTS populations only known to overlap in the Gulf of Oman, the most parsimonious hypothesis is that these lineages diverged in allopatry. However, while sea level fluctuations are a likely driver of divergence among the Pacific and Indian Ocean COTS lineages [11], there are no emergent land barriers (such as the Sunda and Sahul Shelves) in the Indian Ocean, indicating that other processes must be driving diversification in this region.

**Figure 3:**
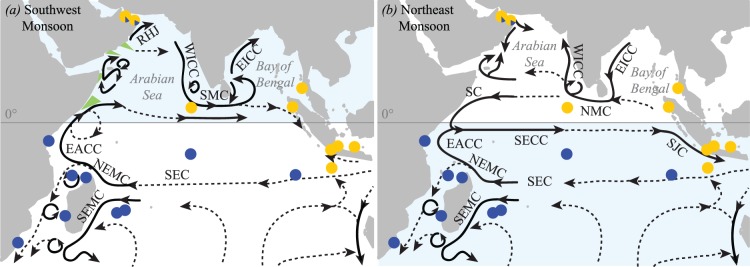
Schematic representation of the Indian Ocean surface circulation. *(a)* During the southwest (July/August) and *(b)* northeast (December/January) monsoon after Schott and McCreary [67], in relation to crown-of-thorns starfish sampling locations (yellow circles: NIO sister-species, blue circles: SIO sister-species). Blue shaded areas indicate the area in which COTS larvae would likely be released according to season. Green wedges in *(a)* are upwelling areas. Current branches indicated are the South Equatorial Current (SEC), Southeast and Northeast Madagascar Current (SEMC and NEMC), East African Coast Current (EACC), Somali Current (SC), Ras al Hadd Jet (RHJ), West and East Indian Coast Current (WICC and EICC), Southwest and Northeast Monsoon Current (SMC and NMC), South Java Current (SJC).

The present distributions of the NIO and SIO sister-species are largely, but not entirely, restricted to the two main current systems to the north and south of the equator, respectively. The Indian Ocean circulation is characterised by strong, seasonal monsoonal current systems and upwelling patterns in the north, whereas an equatorial gyre dominates the tropical southern half (Fig. 3; [31]). The planktonic larvae of COTS display negative geotactic behaviour, i.e. after hatching they swim to the surface and remain there until the late brachiolaria stage (the last stage of their larval cycle before settling; [21]). As such, ocean surface currents are likely to have an important impact on their dispersal, and changes in these currents can be expected to strongly affect the connectivity between populations. It is therefore possible that the divergence of the two species is based on these currents, as lowered sea levels of the Plio-Pleistocene glacial periods are accompanied by pronounced changes in global climate that can have profound impacts on ocean circulation.

Additional insights into the divergence of the Indian Ocean COTS species can be gained from examining the divergence of the major clades within each of these two sister-species. The close timing of the intraspecific divergence (113–139,000 years ago) of the two clades suggests these could have been initiated by one single climatic event. Global sea levels also dropped 120 m below their current level before the onset of the last interglacial (30,000 years ago, isotopic stage 6; [32]). There is strong evidence that during glacial periods, the northern Indian Ocean monsoonal system would have been altered – the seasonal southwest (SW) monsoon being weaker whereas the strength of the northeast (NE) monsoon would have increased [33] in comparison to present-day interglacial patterns (Fig. 3). As suggested by Pollock [34] when investigating interspecific patterns of diversification in spiny lobsters, weaker oceanic circulation could have increased the retention of larvae in the Arabian Sea, thus promoting the diversification of the west and east clades in the NIO sister-species.

In the SIO sister-species, we also detected a western and eastern-origin clade (Fig. 3). Changes in surface circulation resulting from sea-level fluctuations may also have restricted the distribution of COTS in the southern Indian Ocean. However, in this area, past changes in circulation patterns are comparatively poorly documented and still debated. Hutson [35] suggested that intensified westerly winds would have hindered the penetration of the South Equatorial Current and the Northeast Madagascar Current along the southeast coast of Africa (Fig. 3), which could have led to the retention of larvae between the continent and Madagascar, and the subsequent diversification of these populations from other populations of the SIO sister-species. Although more recent findings suggest that temperature and flow in this area were stable for the last 150,000 years, changes in upwelling and eddy formation may still have occurred [36]. The exact location of divergences among the two southern Indian Ocean clades remains unclear, although the central position of the Cocos Keeling haplotypes in the minimum spanning tree might indicate that this area possibly has acted as a refugium (*prov27* in Fig. 1a), although more data would be required to test this hypothesis.

The substantial evidence in favour of the impact of surface circulation changes on population connectivity and subsequent intraspecific divergence provides some support in favour of similar dynamics having acted in the separation process of the two species. However, at this point there is no evidence to suggest anything more specific than that these currents might have helped to maintain the isolation of these species following their divergence. Comparative studies on a broad range of taxa in this region could help clarify the processes driving diversification.

### Intraspecific Population Structure

Despite being closely related and ecologically similar sister-species, there were pronounced differences in the genetic structure of the two COTS Indian Ocean lineages. The NIO sister-species was characterized by strong genetic structure with three regional groupings comprised of western, central and eastern Indian Ocean populations (Table 2). An asymmetric pattern of connectivity was detected between these regions where both western and eastern populations feed into those of the central Indian Ocean (model M_3_ in Fig. 2, Table 5), suggesting that the latter is a dispersal sink. In contrast, while the SIO sister-species has significant genetic structure, it is much less pronounced in this species, suggesting higher levels of connectivity across a similar geographic range. Bayesian skyline plots indicate population expansion in both species, suggesting non-equilibrium dynamics, although there was a very large variance to those estimates. On the other hand, other demographic statistics (Fu’s *F*_S_, Tajima’s *D* and Ramos-Onsins *R*_2_) provide little support for non-equilibrium dynamics in the NIO, while the SIO sister-species showed a strong signature of a recent population expansion (Table 1). This suggests that the differences in genetic structure among NIO and SIO populations may result from regional differences in population stability, either a consequence of abiotic or biotic causes. However, the extent of the deep divergences (13 mutational steps, Fig. 1) separating W. and E. Indian Ocean clades might represent a sampling bias, since we lack samples, for example, from intermediate regions between Kenya and Oman, i.e., the coasts of Somalia and Yemen.

Several hypotheses might explain the observed differences in population genetic structure between the northern and southern Indian Ocean. First, differences in landmass distribution may impact patterns of connectivity. The northern Indian Ocean is bounded by a long coastline on all but its southern margin, and has large numbers of islands in the centre (Maldives) and east (Andamans). While continuous coastline might be expected to yield high genetic connectivity, there are major breaks in reef distributions in the northern Indian Ocean. Upwelling areas off Somalia and Oman, the northern Arabian Sea coast, stretches of the western and eastern coast of Indian, and of the Bay of Bengal lack coral reefs, creating potential barriers to dispersal. In contrast, the southern Indian Ocean has extensive coastlines only on its western and eastern reaches, but the only major breaks in coral distributions are in southern Mozambique and in Madagascar, which are close to the southern end of coral distribution [15] and are therefore unlikely to have a major impact on connectivity in COTS populations.

In addition to landmass impacting distribution of suitable habitat, ocean currents may also play a significant role in creating different levels of genetic structure in the two sister-species. As described above, the main currents in the northern Indian Ocean reverse according to monsoon, which along with strong changes in upwelling patterns, leads to a complex current system. Although it is untested whether data from the Pacific sister-species can be extrapoalted to its other sister-species, COTS larvae are released during a summer spawning season [6] at higher latitudes in the Pacific Ocean (>10°N or S). This period corresponds to the SW monsoon (Fig. 3a) in the northern Indian Ocean, when currents potentially facilitate transport of larvae from the western to central Indian Ocean, consistent with the analysis of gene flow (model M_3_ in Fig. 2). Although direct data on spawning times for populations near the equator are rare, again, data from the Pacific Ocean if extrapolated would suggest there is no discrete spawning season [6]. Thus, movement of larvae from east to central Indian Ocean might occur outside the SW monsoon, when currents flow from east to west (Fig. 3b), with larvae from both the western and eastern Southern Indian Ocean able to reach the Maldives with no or few stepping-stones. Similarly, during the SW monsoon and NE monsoon, the long pelagic larval duration (three to four weeks in the Pacific; [21]) would enable larvae to travel 1200 km on the predominant currents (Fig. S4; [37]), thus reaching the Maldives from respectively Oman or Aceh either directly or within two generations using a stepping-stone (e.g., western Indian coast or Sri Lanka, respectively), resulting in higher connectivity in the SIO.

In the southern Indian Ocean, the consistent gyre would theoretically enable circulating larvae from east to west and vice-versa throughout the year, independent of the spawning time, although larvae from the SIO sister-species are thought to be released during the Austral summer [38]. However, high gene flow was observed in the SIO sister-species, might suggest either modern or (recent) past high connectivity even among extremely isolated populations. Indeed, the Cocos Keeling Islands are separated from their closest downstream neighbour, the Chagos Archipelago, by 2700 km, and the latter from the Seychelles and Rodrigues by another 1600 km. Travelling such large distances in the open ocean far exceeds COTS pelagic larval duration in normal conditions [21]. However, COTS larvae from the Pacific sister-species have been found to extend their developmental period to seven weeks in marginal food regimes [39], although the occurrence of a facultative teleplanic larva remains to be confirmed [6]. Productivity is generally much higher in the northern Indian Ocean, with areas of high productivity (>130 gC.m^−2^) being distributed over a far greater proportion of the northern Indian Ocean (generally associated with the continental margins) than in the southern Indian Ocean (Fig. S5) [40]. As such, low primary productivity in the southern Indian Ocean might result in extended larval durations and higher connectivity, consistent with our results of lowered levels of genetic structure observed in the SIO sister-species, despite the greater geographic distances among populations. In contrast, larval duration in the northern Indian Ocean is unlikely to exceed that found in normal conditions due to the high levels of primary productivity, and we hypothesize that the resulting shorter larval durations contribute to the stronger genetic structure observed in the NIO sister-species.

The presence of a few individuals from the SIO sister-species in populations of the NIO sister-species is quite intriguing (Oman; Fig. 1a). These individuals do not appear to have dispersed into the area during a single founder event, as their haplotypes do not cluster together in the minimum spanning tree (Fig. 1c), suggesting multiple dispersal and colonization events. As no individuals from the SIO sister-species are found in the Maldives, the most likely source of propagules would be the east African coast. Yet the strong upwelling conditions and eddies that accompany the SW monsoon (Fig. 3a) appear to be unsuitable for the transport of larvae from this area to Oman [41]. During the NE monsoon, when populations in the higher latitudes of the southern Hemisphere are most likely to spawn, the southward flowing Somali Current should also hamper the northward dispersal of larvae (Fig. 3b). Although such oceanographic barriers to dispersal should prevent larval crossing, it is clear that occasionally a few propagules are transported against expectations [41]. As Glynn [41] suggested for tropical species in this area, these may represent ephemeral populations that experience brief periods of invasion and extinction.

### Conclusions

Although previously considered a single taxon, northern and southern Indian Ocean populations of *Acanthaster planci* represent genetically distinct sister-species. Differences in genetic structure between them likely result from the interplay of ocean circulation patterns, primary productivity, and proximity to land, all of which combined impact the distribution of available habitat and larval duration. While results clearly indicate that these species are on different evolutionary trajectories, whether this differentiation has led to changes in their biology requires further investigation. It is conceivable that different selective pressures are acting on individuals from the NIO and SIO sister-species, with longer larval phases and better larval dispersal capabilities possibly being selected for in the latter. As the general consensus today is that outbreaks are at least to some extent caused by the effects of primary productivity on larval survival [6]–[8], such differential selection could have far-reaching consequences for differences in outbreak ecology between the Southern and Northern Indian Ocean sister-species, a phenomenon that merits further investigation.

The results of this study also emphasize the importance of conducting further genetic studies of coral reef-associated organisms in the Indian Ocean. There is very little population genetic information available from this ocean [42], yet there is a strong need for more research to increase the overall state of knowledge [43] and devise appropriate conservation strategies [44], [45]. By identifying genetic breaks between and within species as well as exploring the connectivity between populations [46], [47], molecular studies such as this one can not only increase our understanding of the biology of individual organisms, but also contribute to identifying conservation targets, and form the basis for biogeographical classifications and future monitoring [25], [48].

## Materials and Methods

### Sampling and Sequencing

COTS samples were collected by SCUBA and snorkel from 18 sites in the Indian Ocean between 1990 and 2010 (Fig. 1, Table S1). We excluded samples from the southeastern Indian Ocean (Western Australia), as these populations have been previously shown to belong to the Pacific sister-species [10]. We sampled pyloric caeca [11], gonads [17] and/or tube feet. Tissue samples were stored as soon as possible after collection, either at −80°C for the pyloric caeca [11], or in ethanol (>80%), DMSO buffer [49] and on FTA paper (Whatman) for the gonads and tube feet. The DNA was extracted from the pyloric caeca using a MagAttract 96 DNA Plant Core Kit (Qiagen) according to the manufacturer’s manual DNA purification protocol, with the following initial steps: the tissue was manually ground in a 1.5 ml Eppendorf tube after freezing in liquid nitrogen, then incubated at 35°C for an hour in RLT lysis buffer (Qiagen), vortexed at full speed for 20 s, and centrifuged at 8000×*g* for 5 min. DNA was extracted from the other tissues (gonads, and tube feet) using a DNeasy Tissue Kit (Qiagen) according to the manufacturer’s protocol.

A DNA fragment containing the putative mitochondrial control region (CR) and the 5′ end of the adjacent 16S rRNA gene [50] was amplified with the following primers: COTS-CR-F15635 5′-CAAAAGCTGACGGGTAAGCAA-3′ and COTS-CR-R114 5′-TAAGGAAGTTTGCGACCTCGAT-3′. DNA sequencing was performed using the PCR reverse primer, and the following internal forward primer: COTS-CR-seqIO-F15749 5′-GCTTGTGTTCACGGGAAAGC-3′. Cytochrome Oxidase subunit I (COI) sequences from Vogler et al. [10] with additional samples from the Chagos Archipelago (Table S1) were also used. The sequences were assembled using CodonCode Aligner (http://www.codoncode.com/aligner) and aligned in Seaview v4.2 [51] using the built-in MUSCLE software [52]. All new sequences were deposited in the EMBL nucleotide database (see Table S1 for accession numbers).

### Divergence Times and Demographic Patterns

As the CR sequences could not be aligned unambiguously between the Southern Indian Ocean (SIO) and the Northern Indian Ocean (NIO) sister-species, the timing of their divergence was estimated using the COI dataset (Table 1, Table S1). The net divergence *d*_A_ [53] between the two species was calculated using Kimura 2-parameter (K2P) distances estimated in PAUP*4.0b10 [54], approximating divergence times by applying the most accurate COI divergence rates available for echinoderms to *d*_A_ (3.7±0.8%.Myr-1; [18]).

Intraspecific patterns of diversification were investigated by estimating minimum spanning trees in Arlequin v3.5.1.2 [55] for the CR sequences of both SIO and NIO, based on pairwise differences and re-drawn with Adobe Illustrator. To assess the robustness of the signal in the minimum spanning trees, we also constructed split graphs in SplitsTree v4.11.3 [56] using the NeighborNet method, which allow detecting incongruences in the signal and alternative phylogenetic histories.

Because the minimum spanning trees revealed a deep internal split, separating two clades in each species (Fig. 1), we estimated the net divergence *d*_A_ [53] between these clades using the CR dataset (Table S1), as the COI sequences did not offer the necessary resolution. After inferring the best-fit nucleotide evolution model using the Akaike Information Criterion as implemented in jModelTest v0.1.1 ([57]; TPM1uf+I+G for the NIO sister-species, TrN+I+G for the SIO sister-species), *d*_A_ was estimated for the maximum likelihood distances calculated in PAUP*4.0b10 [54].

Since there are no mutation rates available for echinoderm CR sequences, we also used a concatenated COI-CR dataset to calculate the time to the most recent common ancestor *T*_MRCA_ of both the NIO and SIO sister-species, by estimating Bayesian skyline plots in BEAST v1.5.4 [58], [59]. We set a strict clock on COI since preliminary tests showed a clocklike behaviour of the data could not be rejected (zero value of uncorrelated relaxed lognormal clock standard deviation within 95% highest posterior density interval). We used a substitution rate of 1.85±0.4%.Myr^−1^ (normal distribution) in order to incorporate the uncertainty on this rate from the literature [18], and estimated the CR uncorrelated relaxed lognormal clock from COI (see Table S2 for settings).

These Bayesian skyline analyses allowed us to explore the demographic patterns within each of the sister-species, comparing these to statistics that have the ability to detect signatures of recent population expansions: Fu’s *F*_S_ [60] and Tajima’s *D* [61], both calculated using Arlequin v3.5.1.2 [55; 50,000 replicates], as well as Ramos-Onzins *R*_2_ [62], estimated using the R package pegas v0.3–1 ([63]; 10,000 replicates). All these demographic summary statistics were estimated at the species level with the COI dataset, and at the species, clade and population level with CR.

### Spatial Genetic Structure and Migration Patterns

All population level statistics were performed on the CR dataset using Arlequin v3.5.1.2 [55], unless stated otherwise. We calculated standard measures of genetic diversity (haplotype frequencies, haplotype diversity *h*_D_ and nucleotide diversity *π*) for each population and sister-species (CR and COI), as well as pairwise Φ_ST_s between population pairs within each sister-species (50,000 random replicates, standard Bonferroni correction for multiple tests). We also used a Mantel test (100,000 permutations) to determine the relationship between genetic and geographic distances within each sister-species following the method recommended by Rousset [64] for populations in a two-dimensional model, i.e. testing the regression of population pairwise Φ_ST_/(1− Φ_ST_) against the natural logarithm of geographic distances [64]. We then used analyses of molecular variance (AMOVA) to identify regional patterns of genetic differentiation (locus-by-locus AMOVA, 50,000 replicates). We tested several different combinations of groups of populations. These groups were based on geography and published regional provinces (Marine Ecoregions of the World; [19]), with the aim to determine which combination explained the most genetic variation among groups.

In order to understand the connectivity between the regional groups identified by the AMOVA analyses (Table 2), we estimated migration rates and effective population sizes with Migrate v3.1.6 [65], using the Control Region dataset and a Bayesian search strategy as recommended by Beerli [66]. We established the most likely mutation model available in Migrate by using PAUP*4.0b10 [54] to estimate parameters for site rate variation and the transition/transversion ratio, and performed several exploratory runs to determine appropriate priors (Table S3). To explicitly evaluate the performance of different migration models, ranging from panmixia to a full migration matrix (Fig. 2), we ran the analyses with the following heating scheme: [1 1.5 3 10,000] (1,000,000 generations, 32 replicates). This scheme allowed the approximation of marginal likelihoods using thermodynamic integration and hence the estimation of Bayes Factors to compare the performance of different models [20].

## Supporting Information

Figure S1 **NeighborNet analyses of the (a) Northern and (b) Southern Indian Ocean sister-species.** The two main clades within each species are highlighted, and the central Cocos Keeling Island haplotypes in the E_SIO_ clade are surrounded by a grey box.
(PDF)

Figure S2 **Bayesian skyline plots for the (a) Northern and (b) Southern Indian Ocean sister-species.** Black lines are an estimate of effective population size as a function of time, grey lines indicate the 95% upper and lower highest posterior probability interval.
(PDF)

Figure S3 **Genetic distance Φ_ST_/(1−Φ_ST_) as a function of the natural logarithm of geographic distance (in km) for the (a) Northern and (b) Southern Indian Ocean sister-species.**
(PDF)

Figure S4 **Current direction and velocity during the peak of (a) the Southwest Monsoon (January mean from 1993 to 2009) and (b) the Northeast Monsoon (July mean from 1993 to 2009).** Arrow colour indicates direction of flow (westward: blue, eastward: red), arrow length and plot background colour indicate current velocity in meters per second. Data obtained from and plots constructed using Ocean Surface Current Analysis – Real time: http://www.oscar.noaa.gov/(Bonjean and Lagerloef 2002).
(PDF)

Figure S5 **Areas of primary productivity higher than 130 gC/m^−2^.** In grey; modified from Reid et al., 2006, data for 1998–99 [not an El Niño year] after NASA SeaWiFS.
(PDF)

Table S1 **Sampling locations of crown-of-thorns starfish individuals.** With coordinates (decimal degrees), collector or reference, number of Control Region (CR) sequences (*n*_CR_) and of Cytochrome Oxidase I (COI) sequences (*n*_COI_) per clade and location, and EMBL accession numbers (in grey are EMBL accession numbers from Vogler et al. (2008)). Locations preceded by an asterisk are represented in both Indian Ocean sister-species, locations preceded by a dash are shared with the Pacific sister-species.
(PDF)

Table S2 **Run conditions for the BEAST Bayesian Skyline analysis for both the Northern and the Southern Indian Ocean sister-species.**
(PDF)

Table S3 **Run conditions for the Migrate analyses (Control Region dataset) for the Northern (NIO) and Southern Indian Ocean (SIO) sister-species.**
(PDF)

Table S4 **Pairwise Φ_ST_ values for the (a) Northern and (b) Southern Indian Ocean sister-species.**
(PDF)
