# Supplementary figures and images for: Phylogeography of the Crown-of-Thorns Starfish in the Indian Ocean
Source: PLoS One. 2012 Aug 21;7(8):e43499. doi: 10.1371/journal.pone.0043499 (PMC3424128; doi:10.1371/journal.pone.0043499)

**(a) Northern Indian Ocean**

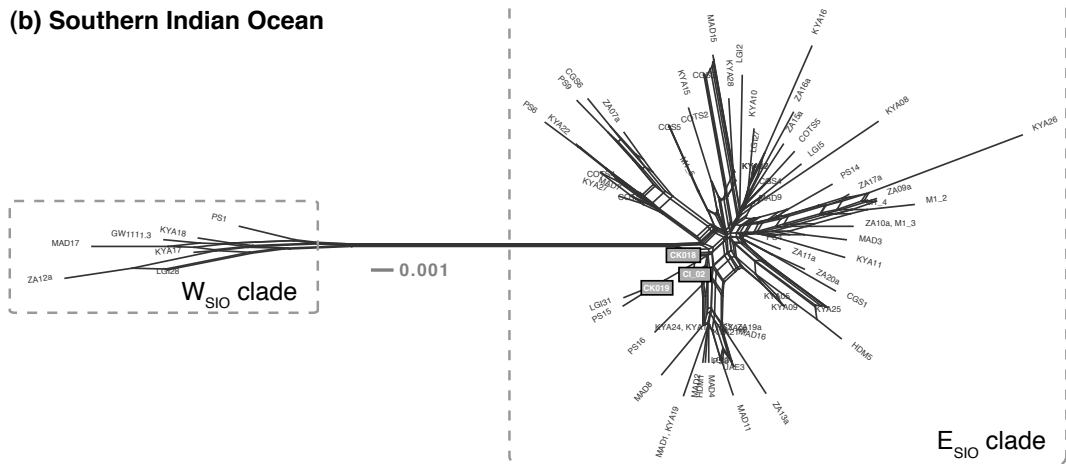

Supplement: Figure S1 — NeighborNet analyses of the (a) Northern and (b) Southern Indian Ocean sister-species. The two main clades within each species are highlighted, and the central Cocos Keeling Island haplotypes in the ESIO clade are surrounded by a grey box. (PDF) [file pone.0043499.s001.pdf]

**Figure S2.**

**Northern Indian Ocean**

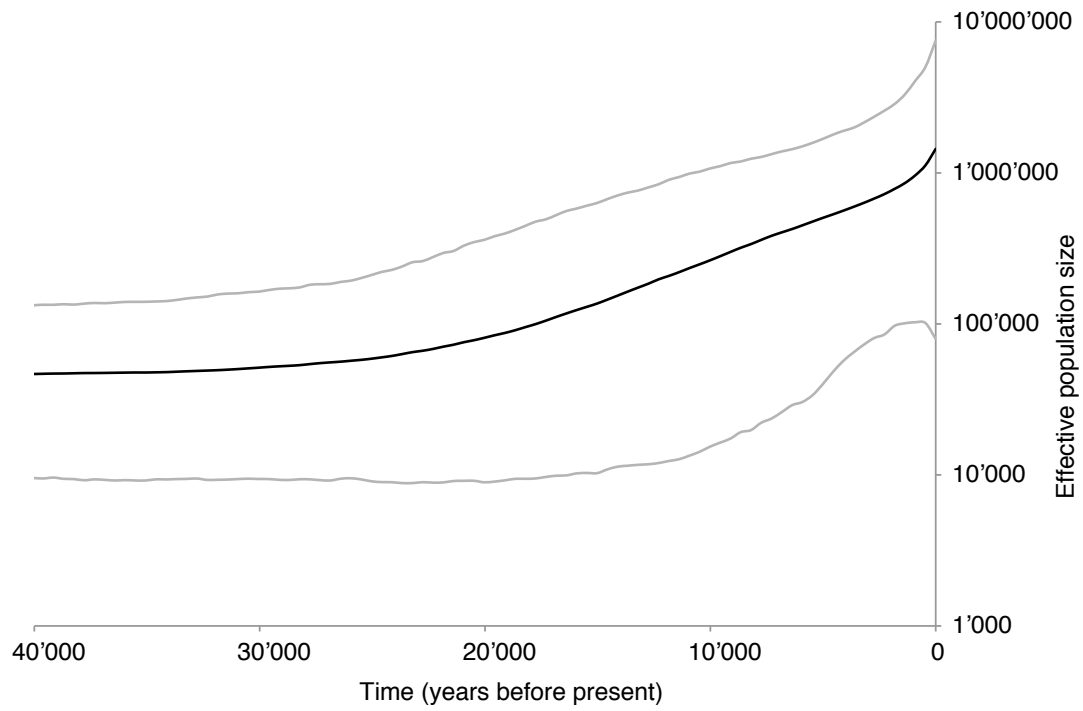

**(b) Southern Indian Ocean**

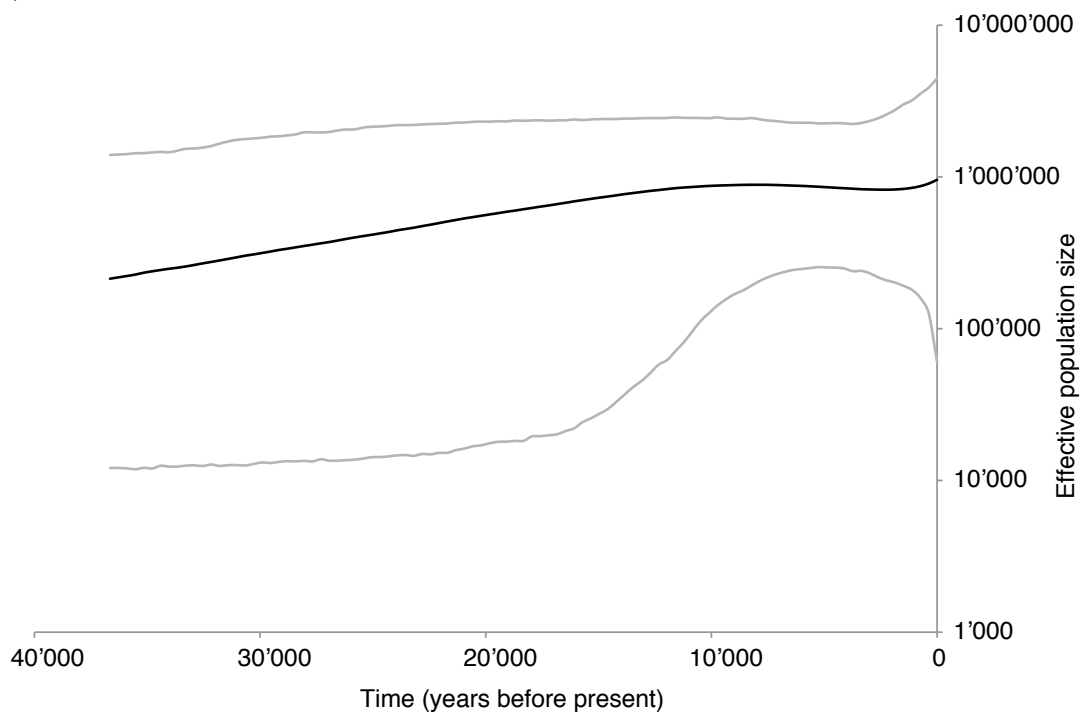

Supplement: Figure S2 — Bayesian skyline plots for the (a) Northern and (b) Southern Indian Ocean sister-species. Black lines are an estimate of effective population size as a function of time, grey lines indicate the 95% upper and lower highest posterior probability interval. (PDF) [file pone.0043499.s002.pdf]

**Figure S3.**

**(a) Northern Indian Ocean**

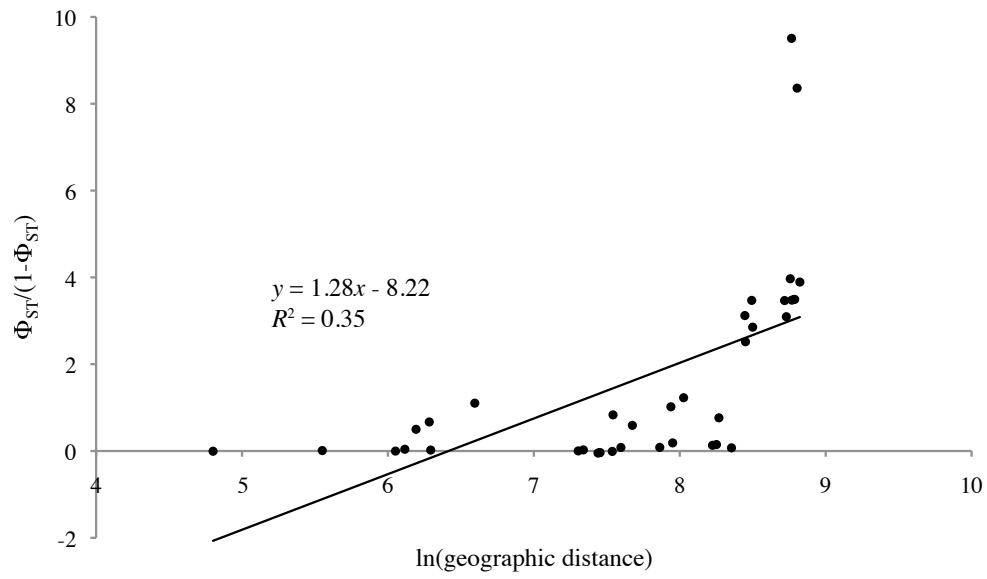

**(b) Southern Indian Ocean**

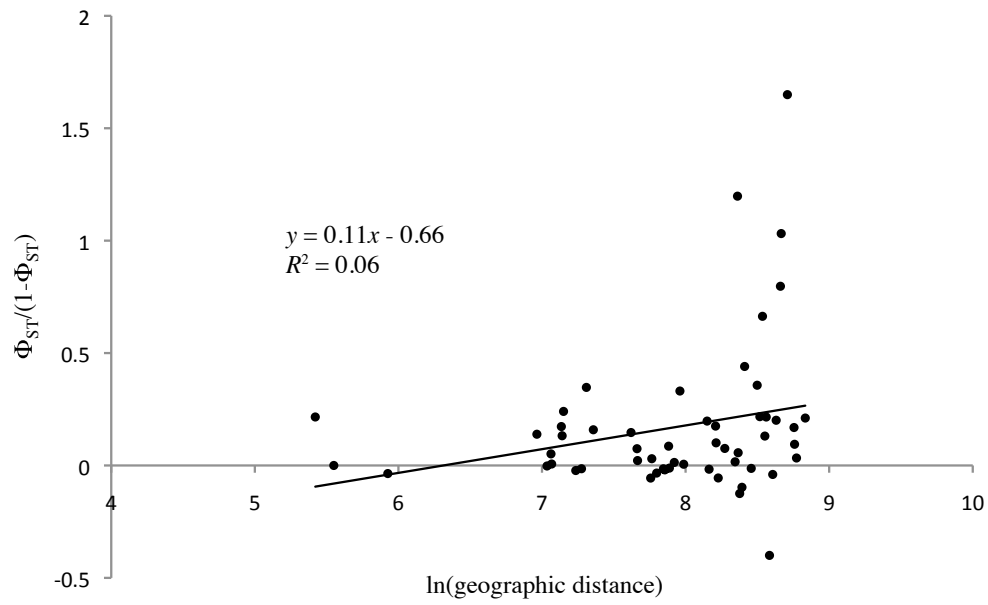

Supplement: Figure S3 — Genetic distance ΦST/(1−ΦST) as a function of the natural logarithm of geographic distance (in km) for the (a) Northern and (b) Southern Indian Ocean sister-species. (PDF) [file pone.0043499.s003.pdf]

Figure S5.

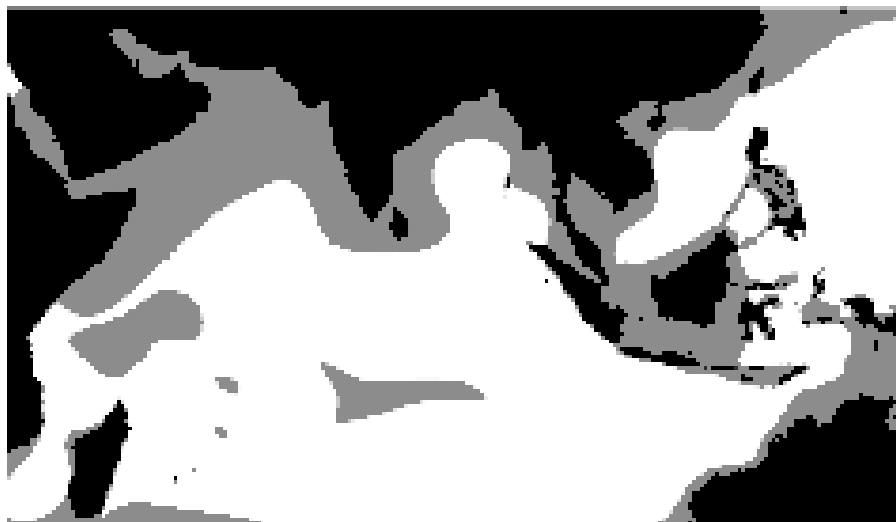

Supplement: Figure S5 — Areas of primary productivity higher than 130 gC/m−2. In grey; modified from Reid et al., 2006, data for 1998–99 [not an El Niño year] after NASA SeaWiFS. (PDF) [file pone.0043499.s005.pdf]
