# Supplementary material for: Phylogeography of the Crown-of-Thorns Starfish in the Indian Ocean
Source: PLoS One. 2012 Aug 21;7(8):e43499. doi: 10.1371/journal.pone.0043499 (PMC3424128; doi:10.1371/journal.pone.0043499)

Figure S4.

(a) Southwest Monsoon: July Mean (1993-2009) Ocean Surface Currents (meter/sec)

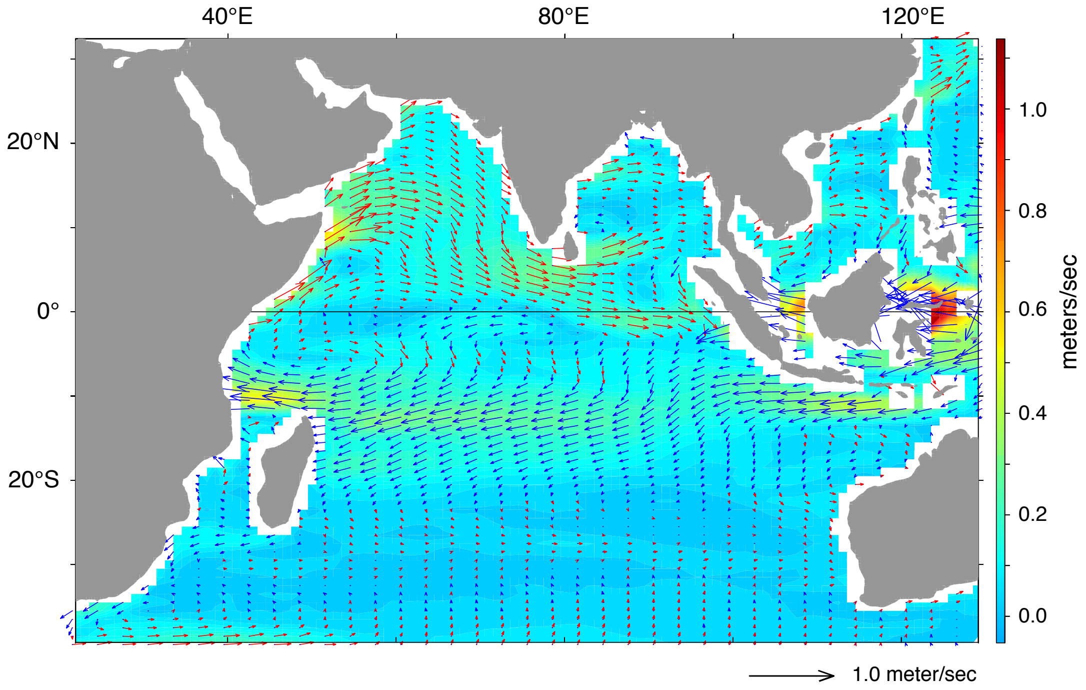

(b) Northeast Monsoon: January Mean (1993-2009) Ocean Surface Currents (meter/sec)

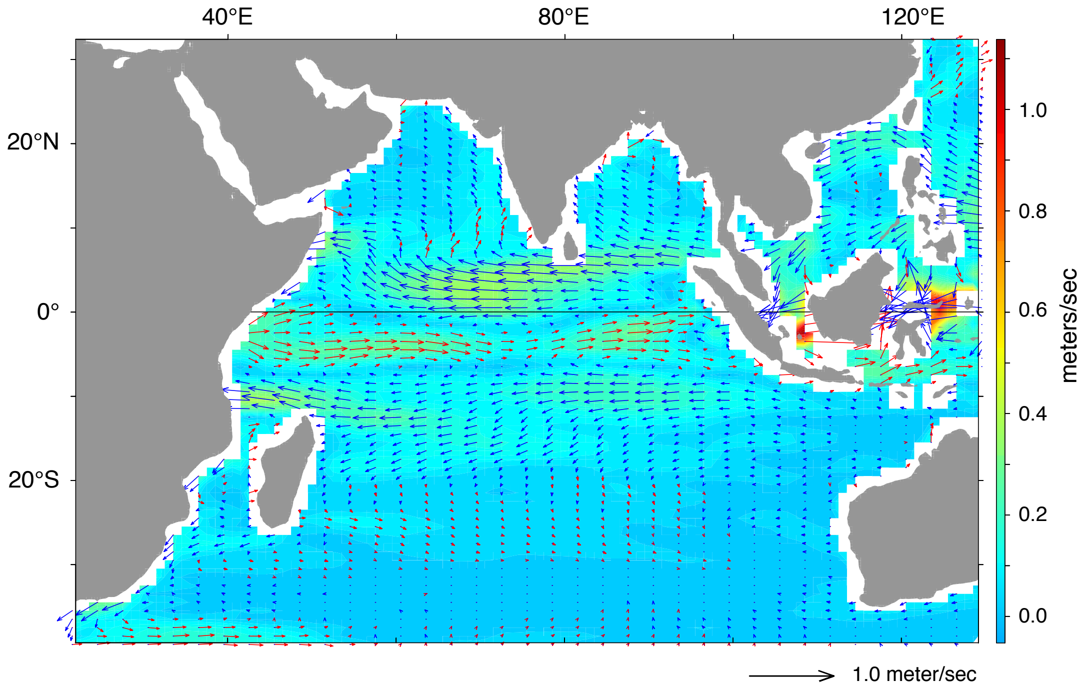

Supplement: Figure S4 — Current direction and velocity during the peak of (a) the Southwest Monsoon (January mean from 1993 to 2009) and (b) the Northeast Monsoon (July mean from 1993 to 2009). Arrow colour indicates direction of flow (westward: blue, eastward: red), arrow length and plot background colour indicate current velocity in meters per second. Data obtained from and plots constructed using Ocean Surface Current Analysis – Real time: http://www.oscar.noaa.gov/(Bonjean and Lagerloef 2002). (PDF) [file pone.0043499.s004.pdf]
