# Supplementary material for: Phylogeography of the Crown-of-Thorns Starfish in the Indian Ocean
Source: PLoS One. 2012 Aug 21;7(8):e43499. doi: 10.1371/journal.pone.0043499 (PMC3424128; doi:10.1371/journal.pone.0043499)
Supplement: Table S1 — Sampling locations of crown-of-thorns starfish individuals. With coordinates (decimal degrees), collector or reference, number of Control Region (CR) sequences (n CR) and of Cytochrome Oxidase I (COI) sequences (n COI) per clade and location, and EMBL accession numbers (in grey are EMBL accession numbers from Vogler et al. (2008)). Locations preceded by an asterisk are represented in both Indian Ocean sister-species, locations preceded by a dash are shared with the Pacific sister-species. (PDF) [file pone.0043499.s006.pdf]

Table S1.

Location	Latitude	Longitude	Sampled by / reference	n_{CR}	EMBL CR	n_{COI}	EMBL COI
**Southern Indian Ocean**				**95**		**57**	
*UAE	25.323	56.382	G. Kirkwood, C. Vogler	2	HE608371-72	2	FM174593-94
*Oman	23.828	58.143	C. Vogler	2	HE608373-74	2	FM174595-96
Réunion	-21.115	55.536	S. Uthicke	5	HE608326-30	5	FM174629-33
Mauritius	-20.348	57.552	Benzie 1999	4	HE608355-58	4	FM174634-38
Kenya	-4.053	39.673	C. Vogler	24	HE608331-54	23	FM174597-619
South Africa	-27.915	32.564	Benzie 1999	12	HE608359-70	9	FM174620-28
Mayotte	-12.724	45.149	K. Gérard	21	HE608375-95	-	-
Nth Madagascar	-12.891	48.604	K. Gérard	11	HE608396-406	-	-
Sth Madagascar	-22.075	43.242	C. Sheppard	2	HE608407-08	-	-
Chagos	-6.638	71.314	C. Sheppard	6	HE608409-14	6	HE608510-15
Cocos Keeling Islands	-12.164	96.871	L. Illidge-Evans, Benzie 1999	6	HE608320-25	6	FM174639-44
**Northern Indian Ocean**				**95**		**48**	
*UAE	25.323	56.382	G. Kirkwood, C. Vogler	15	HE608415-29	10	FM174574-83
*Oman	23.828	58.143	C. Vogler	9	HE608430-38	9	FM174584-92
Maldives	3.203	73.221	Benzie 1999	17	HE608439-55	13	FM174561-73
Christmas Island	-10.447	105.690	L. Illidge-Evans	3	HE608487-89	3	FM174547-49
Aceh	3.954	96.649	P. Barber	15	HE608472-86	-	-
Thailand	7.580	98.522	Benzie 1999	16	HE608456-71	11	FM174550-60
#Pulau Seribu	-5.711	106.597	Benzie 1999, P. Barber	12	HE608490-501	2	FM174545-46
#Karimunjawa	-5.835	110.439	P. Barber	5	HE608505-09	-	-
#Krakatau	-6.047	105.549	P. Barber	3	HE608502-04	-	-
