# Supplementary material for: Phylogeography of the Crown-of-Thorns Starfish in the Indian Ocean
Source: PLoS One. 2012 Aug 21;7(8):e43499. doi: 10.1371/journal.pone.0043499 (PMC3424128; doi:10.1371/journal.pone.0043499)
Supplement: Table S2 — Run conditions for the BEAST Bayesian Skyline analysis for both the Northern and the Southern Indian Ocean sister-species. (PDF) [file pone.0043499.s007.pdf]

Table S2.

	COI	CR
Mutation model	SDR06	HKY+G+I
Molecular clock model	Strict	Relaxed uncorrelated logarithmic
Rate	1.85±0.4%.Myr^{-1}	Estimated from COI
Tree prior	Bayesian skyline	
Number of groups	10	
Skyline model	Piecewise-linear	
Length of chain	10'000'000	
Burn-in	1'000'000	
