# Supplementary material for: Phylogeography of the Crown-of-Thorns Starfish in the Indian Ocean
Source: PLoS One. 2012 Aug 21;7(8):e43499. doi: 10.1371/journal.pone.0043499 (PMC3424128; doi:10.1371/journal.pone.0043499)
Supplement: Table S3 — Run conditions for the Migrate analyses (Control Region dataset) for the Northern (NIO) and Southern Indian Ocean (SIO) sister-species. (PDF) [file pone.0043499.s008.pdf]

Table S3.

	SIO	NIO
Mutation model	F84	F84
Transition/transversion ratio	17.243159	11.329992
Site rate modifier (4 groups)	0.322599 1.744942 4.533686 9.388189	0.323263 1.734373 4.495796 9.299330
Probabilities of site rates	0.602957 0.357582 0.038922 0.000540	0.600390 0.359699 0.039363 0.000548
Prior distribution for mutation-scaled population size	Uniform with range 0.0 to 1.5	Uniform with range 0.0 to 1.0
Prior distribution for mutation-scaled migration rates	Uniform with range 0.0 to 5000	Uniform with range 0.0 to 2000
Proposal distribution for parameters and geneaologies	Slice sampling	Slice sampling
Increment between samples	200	200
Samples per replicate	5'000	5'000
Burn-ins per replicate	100'000	100'000
Replicates	32	32

Table S4.

(a) Northern Indian Ocean

	UAE	Oman	Maldives	Thailand	Aceh	XmasIs	Seribu	Krakatau
Oman	0.010							
Maldives	**0.551**	**0.505**						
Thailand	**0.776**	**0.757**	**0.157**					
Aceh	**0.741**	**0.716**	0.077	0.039				
Christmas Island	**0.893**	0.905	0.433	0.372	**0.454**			
Seribu	**0.777**	**0.756**	0.129	-0.037	0.025	0.401		
Krakatau	**0.799**	0.776	0.116	-0.050	0.002	0.333	-0.005	
Karimunjawa	**0.796**	**0.777**	0.068	0.075	-0.010	0.524	-0.002	0.021

Values in bold were significant after Bonferroni correction: $p<0.00139$.

(b) Southern Indian Ocean

	UAE	Oman	Chagos	CocosIs	SthAfrica	Kenya	Mauritius	Reunion	Mayotte	NthMadagascar
Oman	0.000									
Chagos	0.070	0.149								
CocosIs	0.623	0.508	0.249							
SthAfrica	0.032	0.086	-0.013	0.174						
Kenya	-0.059	0.091	-0.017	0.144	0.013					
Mauritius	0.399	0.263	0.069	0.545	-0.012	0.079				
Reunion	0.116	0.178	-0.059	0.306	-0.035	-0.015	0.177			
Mayotte	-0.108	0.054	0.005	0.167	0.021	-0.002	0.137	-0.015		
NthMadagascar	-0.143	0.016	-0.020	0.177	0.029	-0.023	0.117	0.006	-0.037	
SthMadagascar	-0.042	-0.667	0.165	0.444	0.148	0.128	0.258	0.194	0.122	0.049

Values in bold were significant after Bonferroni correction: $p<0.0009$.
