# Supplementary material for: Phylogeography of the Crown-of-Thorns Starfish in the Indian Ocean
Source: PLoS One. 2012 Aug 21;7(8):e43499. doi: 10.1371/journal.pone.0043499 (PMC3424128; doi:10.1371/journal.pone.0043499)
Supplement: Table S4 — Pairwise ΦST values for the (a) Northern and (b) Southern Indian Ocean sister-species. (PDF) [file pone.0043499.s009.pdf]

Table S4.

(a) Northern Indian Ocean

	UAE	Oman	Maldives	Thailand	Aceh	XmasIs	Seribu	Krakatau
Oman	0.010							
Maldives	**0.551**	**0.505**						
Thailand	**0.776**	**0.757**	**0.157**					
Aceh	**0.741**	**0.716**	0.077	0.039				
Christmas Island	**0.893**	0.905	0.433	0.372	**0.454**			
Seribu	**0.777**	**0.756**	0.129	-0.037	0.025	0.401		
Krakatau	**0.799**	0.776	0.116	-0.050	0.002	0.333	-0.005	
Karimunjawa	**0.796**	**0.777**	0.068	0.075	-0.010	0.524	-0.002	0.021

Values in bold were significant after Bonferroni correction: $p<0.00139$.

(b) Southern Indian Ocean

	UAE	Oman	Chagos	CocosIs	SthAfrica	Kenya	Mauritius	Reunion	Mayotte	NthMadagascar
Oman	0.000									
Chagos	0.070	0.149								
CocosIs	0.623	0.508	0.249							
SthAfrica	0.032	0.086	-0.013	0.174						
Kenya	-0.059	0.091	-0.017	0.144	0.013					
Mauritius	0.399	0.263	0.069	0.545	-0.012	0.079				
Reunion	0.116	0.178	-0.059	0.306	-0.035	-0.015	0.177			
Mayotte	-0.108	0.054	0.005	0.167	0.021	-0.002	0.137	-0.015		
NthMadagascar	-0.143	0.016	-0.020	0.177	0.029	-0.023	0.117	0.006	-0.037	
SthMadagascar	-0.042	-0.667	0.165	0.444	0.148	0.128	0.258	0.194	0.122	0.049

Values in bold were significant after Bonferroni correction: $p<0.0009$.
